# Atorvastatin and simvastatin protects cognitive impairment in an animal model of sepsis

**DOI:** 10.1186/cc13007

**Published:** 2013-11-05

**Authors:** Pedro CB Alexandre, Patricia A Reis, Joana D`Ávila, Flora M de J Oliveira, Fabricio A Pamplona, Luciana D Siqueira, Hugo CC Faria Neto, Fernando A Bozza

**Affiliations:** 1 Laboratorio de Imunofarmacologia, IOC/FIOCRUZ, Rio de Janeiro, Brazil; 2 Instituto D'Or de Pesquisa e Ensina - IDOR, Rio de Janeiro, Brazil; 3 Instituto de Pesquisa Clinica Evandro Chagas - IPEC/FIOCRUZ, Rio de Janeiro, Brazil

## Background

Recently it was shown that a significant proportion of sepsis survivors can develop a transitory or permanent cognitive impairment. Statins have the ability to block the cascade of cholesterol formation by acting on HMG-CoA reductase, reducing the synthesis of endogenous cholesterol. Recently it has been observed that statins have anti-inflammatory properties preventing brain dysfunction in malaria models, reducing the production of brain cytokines, oxidative stress and alterations in the blood-brain barrier. The aim of the present study was to evaluate the ability of statins to reduce neuroinflammation and protect septic animals from neurocognitive damage.

## Materials and methods

Feces were extracted (5 mg/g b.w.) from the large intestine of SW mice and diluted in saline, centrifuged and the supernatant collected and injected into the animals (*n *= 5 to 8/group). Control animals received 0.5 ml saline. Animals were treated at 6, 24 and 48 hours after sepsis induction with imipenem (30 mg/kg b.w., 0.2 ml s.c.) and 1.0 ml saline (s.c.). Statins (Ator and Sinv) were administrated 1 hour before and 6, 24 and 48 hours after the infection (20 mg/kg b.w., p.o.). Mortality was observed for 96 hours and a score of severity evaluated. The inflammatory profile and oxidative damage was determined at 6 and 24 hours. In addition, mice brains were evaluated for microglial activation and BBB dysfunction. After 15 days we analyzed the cognitive damage using the inhibitory avoidance task and Morris water maze.

## Results

No significant difference in survival was observed comparing septic animals treated with antibiotics plus atorvastatin or simvastatin (56%; 53%) with septic animals with only antibiotics (37%). We observed lower levels of proinflammatory cytokines (IL-1, IL-6) and chemokines (KC and MCP-1) when comparing statin-treated animals and nontreated. We also observed a decreased in the oxidative damage in brains 6 hours after sepsis in the treated groups. Finally, statin treatment was able to protect septic animals from cognitive damage including avoidance and spatial memory, both affected in untreated infected mice.

## Conclusions

We can conclude that statins protected septic animals from cognitive damage, reducing neuroinflammation, and adjuvant therapies with statins can be interesting targets for future clinical trials focused on the prevention of long-term cognitive decline in sepsis.

